# Importance of Silicon and Mechanisms of Biosilica Formation in Plants

**DOI:** 10.1155/2015/396010

**Published:** 2015-01-21

**Authors:** Mahbod Sahebi, Mohamed M. Hanafi, Abdullah Siti Nor Akmar, Mohd Y. Rafii, Parisa Azizi, F. F. Tengoua, Jamaludin Nurul Mayzaitul Azwa, M. Shabanimofrad

**Affiliations:** ^1^Laboratory of Plantation Crops, Institute of Tropical Agriculture, Universiti Putra Malaysia, 43400 Serdang, Selangor, Malaysia; ^2^Department of Land Management, Faculty of Agriculture, 43400 Serdang, Selangor, Malaysia; ^3^Laboratory of Food Crops, Institute of Tropical Agriculture, Universiti Putra Malaysia, 43400 Serdang, Selangor, Malaysia

## Abstract

Silicon (Si) is one of the most prevalent macroelements, performing an essential function in healing plants in response to environmental stresses. The purpose of using Si is to induce resistance to distinct stresses, diseases, and pathogens. Additionally, Si can improve the condition of soils, which contain toxic levels of heavy metals along with other chemical elements. Silicon minimizes toxicity of Fe, Al, and Mn, increases the availability of P, and enhances drought along with salt tolerance in plants through the formation of silicified tissues in plants. However, the concentration of Si depends on the plants genotype and organisms. Hence, the physiological mechanisms and metabolic activities of plants may be affected by Si application. Peptides as well as amino acids can effectively create polysilicic species through interactions with different species of silicate inside solution. The carboxylic acid and the alcohol groups of serine and asparagine tend not to engage in any significant role in polysilicates formation, but the hydroxyl group side chain can be involved in the formation of hydrogen bond with Si(OH)_4_. The mechanisms and trend of Si absorption are different between plant species. Furthermore, the transportation of Si requires an energy mechanism; thus, low temperatures and metabolic repressors inhibit Si transportation.

## 1. Introduction

Generally, food security and health concerns are two critical issues, for human life. Due to the population growth, especially in developing countries, and the spread of communicable and noncommunicable diseases in human population, having a flexible agricultural system is more necessary than ever. Agricultural systems are reliable ways to increase food for the humans by using natural resources. To increase the food qualities and quantities, plants should utilize different strategies to overcome the adverse environmental effects. By utilizing genes strategies, plants can increase their resistance against negative environmental impacts. Along with that, scientists made an effort to increase plants tolerance against pathogens. Silicon (Si), as a macroelement, has a vital role in plants cycles. This element is the eighth most common element in nature and the second most common element found in soil after oxygen. One of the main functions of Si is improving the plants growth and yield especially in stress condition. To achieve plant tolerance, Si promotes plant photosynthesis by favourably exposing leaves to light. On the other hand, the role of the macroelement has proven to be in response to different abiotic and biotic stress. Meaningfully, increasing resistance to diseases and pathogens, metal toxicities, salinity and drought stresses are some of the most important functions of this factor. Indeed, protecting plants against extremely high or low temperature needed for nodule configuration, as well as for beneficial effect over the mineral composition and enzyme activities of plants are other advantages of the macroelement [1]. Plant growth depends on several elements existing in the soil. These elements can be categorised into beneficial, essential, and toxic groups [2]. Toxic elements unconstructively affect plant growth, while essential elements display critical roles for all plants in different growth conditions. Beneficial elements are vital for some specific plant species growing under certain growth situations. The beneficial effects of Si on different plant species are well documented. Notwithstanding the above advantages of this factor, some scientists still believe in unnecessary function of Si in plants cycles. In contrast, lack of documented proof conducted researchers to find the proper role of this element in plants. The second group believes Si is an omnipresent and important element of plants and soil. In addition, they observed that Si has many beneficial roles in crop performance and life [3]. For example, results of a study on the function of Si over controlling powdery mildew in cucumber demonstrated this macroelement is able to produce inactive phytoalexins or glycosylated [4], which are activated by infection of Si-treated plants with fungi leading to cell death of fungi [5]. It has been reported that Si is able to increase stress tolerance and decrease membrane damage in tomato (*Solanumly copersicum*) and spinach (*Spinacia oleracea*) [6]. On the other hand, Si helps wheat (*Triticum* spp.) to overcome oxidative damage in pots under drought stress and powdery mildew [7, 8]. Silicon also leads sorghum (*Sorghum bicolor*) to enhance drought tolerance [9], rice (*Oryza sativa*) to enhance sheath blight (*Rhizoctonia solani*) and blast disease resistance [10–12], sugarcane (*Saccharum officinarum*) to decrease susceptibility against eldana saccharina (*Lepidoptera: Pyralidae*) [13], barley (*Hordeum vulgare* L.) [14, 15] and cucumber (*Cucumis sativus*) to increase salt tolerance [14–16], and maize (*Zea mays* subsp.* mays*) to enhance cadmium tolerance, decline aluminium toxicity, and improve efficiency of using water [17–19]. Moreover, the main important role of Si in plants is during exposing to abiotic and biotic stress. The macroelement is able to suppress these stresses in plants, leading to higher plant productivity [20]. To support, fertilisation of paddy soil with Si, which began in 1955 in Japan, increased rice production tremendously [21].

Additionally, according to results of an investigation released, plants treated with Si have strong structure [22]. Moreover, these plants in comparison to control are more resistant against biotic and abiotic stress such as pathogens and metal toxicities, respectively. The results of investigation also strongly confirmed the biosilica formation role of the* serine-rich protein* gene in transgenic* Arabidopsis thaliana* [23].

Generally, the above proof released the necessary function of Si in pant cycles [22]. Most of soils are able to provide sufficient nutrients for plants without any extra fertiliser. However, increasing of plant growth and productivity can be affected by artificially modifying the soil nutrients through fertilisation because plants can obtain necessary nutrients from the fertilised soil. Adding Si as fertilizer to the soil may not provide the same efficiency for all plants and crops in different geographical areas and soil conditions. Hence, genetic modification of plants in order to absorb more Si from the soil and accumulate it in their roots and shoots seems to give more efficient and sustainable results. Cation exchange resulting from hydrogen (H^+^) pumping by the root hairs of plants leads to nutrient uptake because the H^+^ ions relocate the cations (negatively charged particles) that are attached to the soil particles into the plant roots. In this review, we illustrate several physiological, biochemical, and molecular factors that affect Si absorption and biosilica formation mechanisms in plants. Additionally, this review provides useful information regarding a previously discovered novel* serine-rich protein* gene that plays a crucial role in the biosilica formation in plants [24].

## 2. Important Roles of Silicon

### 2.1. Forms of Si in Soil

Each kilogram of soil usually contains Si ranging from 50 to 400 grams. Silicon dioxide (SiO_2_) is the common form of Si in soil. Vermiculite, smectite, kaolin (rich minerals in soils), orthoclase, feldspars, plagioclase (silicates in the form of crystal), amorphous silica, and quartz are the main Si components in most soils structures [25]. Solubility of all the above Si forms is low and biogeochemically immobile. The major soluble forms of Si in the soil are poly- and monosilicic acids [26]; however, monosilicic acid occurs mostly in a feebly adsorbed condition [27] and has low capability to migrate inside the soil [28]. By increasing the monosilicic acid concentration in the soil solution, plants are able to absorb phosphates (P) directly. The amount of monosilicic acid is increased because of chemical resemblance between phosphate and silicate anions causing a competitive reaction in the soil [29]. Insolubility of monosilicic acid decreases slightly through interactions with heavy metals, iron, aluminium, and manganese [30].

Essential soil components are polysilicic acids that commonly influence the physical properties of soils. In contrast, monosilicic acidic adsorbent is chemically immobile and makes colloidal particles [31]. Thus, polysilicic acids interfere with soil structure formation and soil water-holding [32]. In the biochemical processes which occur in the soil, the Si function is highlighted because this element possesses chemical properties that can create molecules with useful biological functions. The main drawback of Si is the disability of this element to form chemical bonds with different types of atoms that are necessary for the chemical versatility of metabolism. This disability is caused by the size and the molecular mass of the Si atom, which leads to limiting interaction with the other atoms and the formation of monotonous molecules.

### 2.2. Silicon and Plants

#### 2.2.1. Variability of Si Contents in Various Plant Species

Among plants, sugarcane (*Saccharum officinarum*), rice (*Oryza sativa*), and wheat (*Triticum* spp.) absorb the largest amount of Si, with 300–700, 150–300, and 50–150 kg Si ha^−1^, respectively [33]. Generally, Si uptake in graminaceous plants is much higher than its uptake in other plant species. For example, rice is a common Si-collector that absorbs Si in active progression [34], as other graminaceous plants do including wheat (*Triticum* spp.) [35], barley (*Hordeum vulgare* L.) [36], ryegrass (*Lolium perenne*) [37], maize (*Zea mays* subsp.* Mays*) [38], and some cyperaceous plants. The majority of dicotyledon plants, such as cucumbers (*Cucumis sativus*), melons, strawberries, and soybeans (*Glycine max* L. Merr) absorb Si inertly [39]. Nonetheless, some plants especially dicotyledon, such as tomatoes (*Solanum lycopersicum*), beans, and other plants, are not able to absorb Si from soil [39–42].

#### 2.2.2. Absorption Forms of Si by Plants

**Figure 1:**
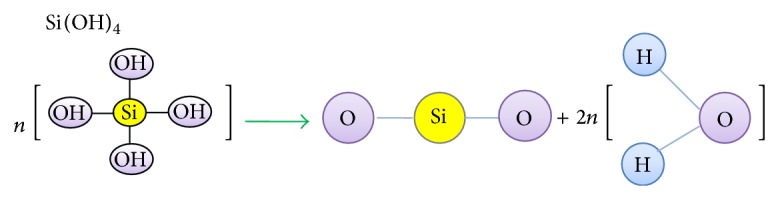
Mono- and polysilicic acids hydration.

Monosilicic acid or orthosilicic acid (H_4_SiO_4_) is the Si forms that are absorbed by plants root [43, 44]. Consequently, Si accumulates in the epidermal tissues, and a layer of cellulose membrane-Si is created when Ca and pectin ions are present [45], which provides protection to the plant [19, 46, 47]. Increasing of Si in the sap of plants leads to Si polymerisation [48], identified as Si gel hydrated with water molecules [49]. The process of mono- and polysilicic acids hydration is as follows (Figure 1).

**Figure 2:**
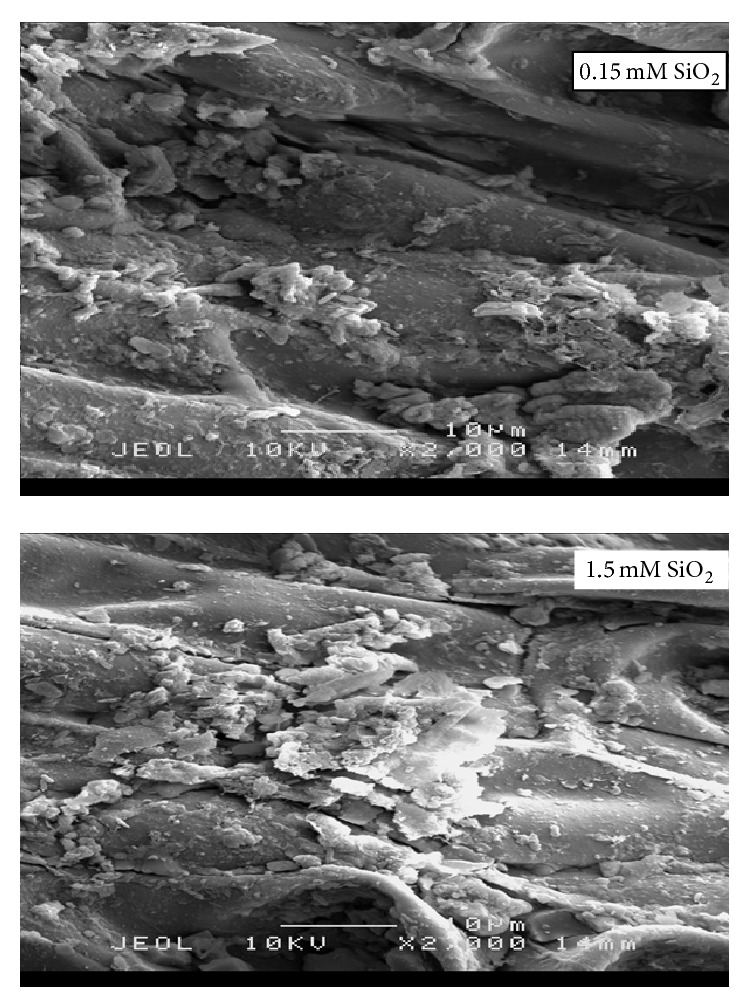
Scanning electron microscopy image of Si absorption by roots of mangrove under different concentrations of SiO_2_ in Hoagland's solution.

Recently, Nurul Mayzaitul Azwa (*personal commun*.) reported that mangrove plants can absorb large amounts of Si from the soil solution (Figure 2). Amorphous silica is the final form of 90% of absorbed and transformed Si in Si-cellulose structures [50]. A nanometre level of biogenic silica is produced as intercell structures [51]. Concentration of Si differs significantly in the shoots and roots of plants, and this extensive variation in different plant tissues is related to differences in the mechanisms of Si uptake and transportation [52, 53]. Nutrient uptake by plants depends on the potential of water and the solubility of elements in the soils. The nutrients uptake pathway is from the soil solution with a higher solute concentration to plant tissues with a lower solute concentration. Although Si is found plentifully in both silicate and oxidase forms in the soil, Si solubility in the soil solution is an obstacle for plant absorption because monosilicic acid is the only form of Si that plants can absorb.

### 2.3. Silicon and Abiotic Stresses

It has been widely reported that Si is able to suppress both physical stress, such as drought, high temperature, UV, loading, and freezing, and chemical stress, including salinity, nutrient imbalance, and metal toxicity [54, 55].

#### 2.3.1. Silicon and Salinity Stress

Salinity stress, a major yield restraining factor in dry and semidry areas, can be repressed by increasing Si [56]. Silicon indirectly reduces the oxidative damage of cucumber tissues under salt stress through the activities of guaiacol peroxidase, ascorbate peroxidase, superoxide dismutase, dehydroascorbate reductase, and glutathione reductase [16]. Oxidative damage in tomato leaves decreases with increasing Si [5], resulting in increased activity of catalase and superoxide dismutase enzymes, increased protein content in the tomato leaves, decreased ascorbate peroxidase enzyme, decreased malondialdehyde concentration, and decreased H_2_O_2_ levels [57]. The alleviative effect of Si over salinity stress has been demonstrated in wheat (*Triticum* spp.) [55, 58], rice (*Oryza sativa*) [55, 59], barley (*Hordeum vulgare* L.) [14, 40, 55, 60], mesquite [61], tomato (*Solanum lycopersicum*) [57, 62], cucumber (*Cucumis sativus*) [63], and maize (*Zea mays* subsp.* mays*) [64, 65]. The roots and shoots of Si-treated rice plants under salinity stress notably improved when compared to control plants [66]. It has been reported that the salt tolerance of mesquite and wheat can be increased significantly after supplying a Si nutrient solution at small amounts [58]. Along with that, the salinity tolerance of hydroponically cultured rice can be increased by adding Si to the nutrient solution [67]. Adding Si decreased the concentration of Na in barley shoots [68] and rice shoots [69].

The positive physiologic effects of silicon on improvement of plants are in conjunction with the endogenous stress responses of plants in different environmental condition [70]. Silicon is able to increase soluble protein content of plants' leaves, which helps plants to overcome salt stress by replacing the lost soluble protein content under salinity stress [16]. Silicon can also increase the antioxidant enzyme activity of superoxide dismutase (SOD), guaiacol peroxidase (GPX), ascorbate peroxidase (APX), dehydroascorbate reductase (DHAR), and glutathione reductase (GR) in plants under salt-stress [14, 16, 68]. The induced oxidative damage by salt can be decreased through decreasing in level of electrolyte leakage percentage (ELP), lipid peroxidation (LPO), and H_2_O_2_ content [16]. This enzymatic protection mechanism helps plants to overcome salinity stress damage [71–73]. A considerable enhancement in the antioxidant enzyme activities in leaves of salt-stressed cucumber by additional Si treatment suggested that Si can be involved in physiological or metabolic cycles of plants [16]. The Si nutrition increased catalase activity significantly in all parts of plants and peroxidase activity in cell wall of plant's shoots [57, 74].

From the physical stand point, Si is able to decrease the plasma membrane permeability in leaf cells of plants which resulted in reducing the lipid peroxidation levels. The Si application of plants under stress condition leads to decreasing lignin content in cell walls of plant's shoots. It was reported that application of Si in canola plants resulted in decreasing Si content in shoot parts of plants by formatting complexes of Si-polyphenol or substitution of Si and lignin [75]. These physical changes in plants' cell wall could facilitate loosening process and promote cell extension, which results in plants' growth under salt stress [74, 76]. The Si protects plants from environmental stress, such as drought and heat, by providing more stable lipids involved in their cell membrane [16, 77].

**Figure 3:**
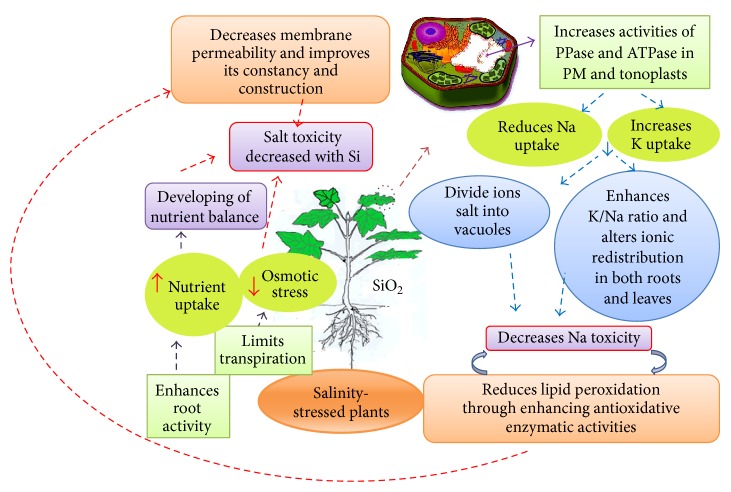
Schematic mechanism of the interaction of Si treatment and salt stressed plants.

It was considered that Si-induced motivation from plasma membrane of roots might increase absorption and transportation of K and decrease the uptake and transportation of Na from the roots to shoots of barley under salinity [60]. On the other hand, Na^+^ ion concentration in canola tissues under salt stress is decreased with Si application. The Si accumulation in the endodermis and cell walls of plants could reduce the Na accumulation in roots and shoots* via* a diminution in apoplastic transportation [74, 78–81]. Plants under salinity stress encounter low water potential from the outside because of the high Na^+^ and Cl^−^ content in the soil and salt deposition in the other plant cellular regions [82]. These ions move to the aerial parts of plants via transpiration, and when Na^+^ and Cl^−^ are at a toxic threshold, different plant tissues can be harshly damaged. The hydrophilic nature of Si can decrease the poisonous levels of saline ions and reduce the osmotic effect of salt stress on the absorption and storage of water by plants. Additionally, Si treatment in plants results in enlarged leaf cells* via* cell wall expansion, which helps the plants to hold more water. Reportedly, Si treated plants grown under saline stress have a larger leaf weight ratio and a smaller specific leaf area than untreated plants, which have smaller leaf surface areas and loss of water transpiration [58]. It has been shown that when salinised plants were treated with Si, their water amounts increased up to 40%. Moreover, the turgor potential of plants treated with both NaCl and Si was 42% higher than that of NaCl-treated plants [62]. Silicon-treated salinised plants showed 17% higher efficiency in using water than untreated plants [16]. This suggests that Si is able to alleviate the harmful effects of salinity stress. Silicon can decrease lipid peroxidation in plants exposed to salinity stress by enhancing the enzymatic and nonenzymatic activities of antioxidants [16, 57]. Silicon application to the plants under salt stress limits the transpiration ratio and increases root activities. Decreases in transpiration lead to reduced osmotic stresses in plant cells and improved root activities. As consequence of root activities, plants can increase the nutrients uptake and decrease salt toxicity. Silicon absorption by plants leads to increased PPase and ATPase activities in vacuoles, which reduces Na^+^ uptake and enhances K^+^ uptake by the cell membrane. Separation of salt ions into the vacuoles and increasing the K^+^/Na^+^ ratio in the cells of the roots and leaves decrease Na^+^ toxicity. Increasing antioxidative enzymatic activities cease electron losses from the lipids in cell membranes, which decreases lipid peroxidation and cell damage. The schematic of the mechanism of the interaction of Si treatment in plants under salinity stress is shown in Figure 3.

#### 2.3.2. Silicon and Heavy Metals

*(1) Silicon and Manganese Toxicity*. The role of Si in suppression of heavy metal toxicity is broadly noted in higher plants. The alleviating role of Si against Mn in the solution culture of barley was first discovered in 1957 [83, 84]. Although Si is not able to affect the whole Mn in barley leaves, it is able to evenly distribute Mn across the entire leaf and does not allow Mn to concentrate in distinct necrotic spots [85]. Additionally, the function of Si in alleviating Mn toxicity has been widely reported in rice [55], pumpkin [20, 86], barley [87], sorghum [88], maize [89], beans [90], soybeans [91], cucumbers [75, 85, 92], and cowpeas [93, 94].

The cation binding ability in the cowpea cell wall can be adjusted by Si [95]. Silicon is able to suppress Mn toxicity either by reducing the soluble apoplastic concentration of Mn in the cell wall or with apoplastic Mn detoxification [93, 94]. The results of a study by Iwasaki et al. show that released Si can cause Mn oxidation in the deposited form* via* relation with apoplast phenolic substances, which results in improving the tolerance of leaves to Mn [93, 94]. Silicon can decrease Mn toxicity by binding the majority of Mn in the cell walls of leaf tissues, and only a small amount of Mn is found in the symplast [96]. Silicon suppresses Mn toxicity in cucumbers by reducing the effects of membrane lipid peroxidation and increasing the enzymatic and nonenzymatic activities of antioxidants [85].

**Figure 4:**
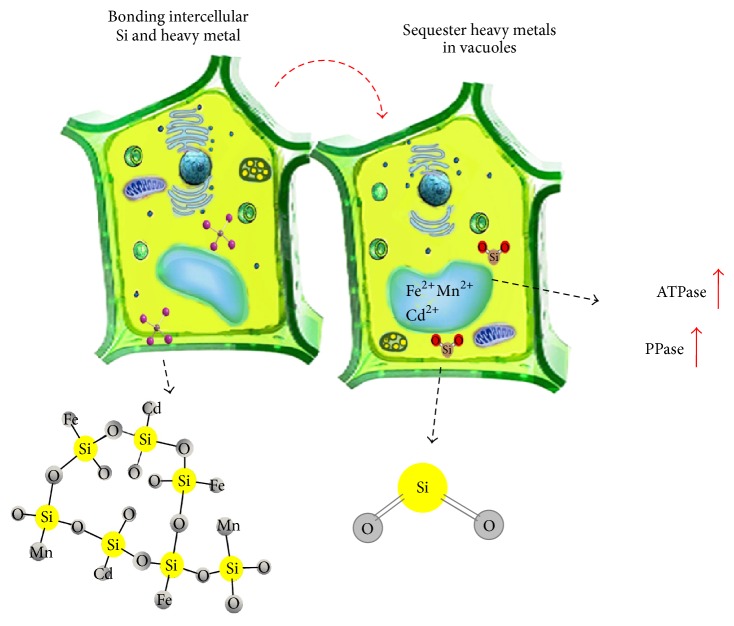
Interaction between intercellular Si and heavy metals.

*(2) Silicon and Cadmium Toxicity*. Silicon can decrease cadmium (Cd) toxicity created by increasing the pH through a detoxification process. It has been reported that Cd uptake in plants is reduced through increasing obtainable Si and raising the pH [97]. The role of Si as a supplementation factor which is effective in decreasing Cd toxicity has been reported in cucumbers [98], maize [99–101], rice [102–104],* Brassica chinensis* [105], and peanuts [106]. Silicon minimises metal ion absorption and limits the transformation of toxic metals between the roots and shoots of rice seedlings grown in Cd [107]. Deposition of Si around the endodermis provides the potential to control Cd apoplastic transportation by physically obstructing the apoplast bypass flow in the root [107]. The Si treatment of maize under Cd stress significantly increased the biomass of the plant by reducing Cd availability and increasing soil pH [17]. The alleviating function of Si on Cd toxicity is not limited to the immobilising role through increased pH of soil, Si also aids in Cd detoxification in maize [17]. Silicon has a similar role in increasing the tolerance of plants to Mn and Cd toxicity by immobilising the metals in the cell walls of the root and inhibiting their transport to the cytosol [93, 94, 96]. These studies have suggested that Si is able to covalently bind with heavy metals and form an unstable silicate form (Figure 4), which subsequently suppresses the toxicity of the metals and is easily degraded to silicon dioxide (SiO_2_). Hence, it can be figured out that Si displays crucial role in intercellular and extracellular parts of plants' cells. The extracellular activities of Si are by limiting penetration of heavy metals into the cytoplasm depending on its Si concentration. Sequestering of heavy metals in vacuoles is the intercellular activity of Si that happened in cytoplasm.

*(3) Silicon and Aluminium Toxicity*. The suppressive effect on aluminium (Al) toxicity in plants by Si treatment and the potential mechanisms of this suppression have been thoroughly investigated. The role of Si in the alleviation of Al toxicity is different between plant species. In this regard, Si can significantly decrease Al toxicity in Zea mays (*Teosinte* L. ssp. Mexicana), barley (*Hordeum vulgare* L.), soybeans (*Glycine max* L. Merr), and sorghum (*Sorghum bicolor*). However, in other species such as wheat (*Triticum aestivum* L.), rice (*Oryza sativa* L.), pea (*Pisum sativum* L.), and cotton (*Gossypium hirsutum*), Si is not effective in decreasing Al toxicity [108]. Additionally, using Si as an alternative detoxification method for Al toxicity has been reported in sorghum (*Sorghum bicolor* L.) [109, 110], tomato (*Lycopersicum esculentum* L.) [111], and barley (*Hordeum vulgare* L.) [112]. It has been documented that Al toxicity containment by Si in soybeans is not stable and depends on the pH [110]. The Al concentration can increase during Si treatment by forming hydroxyl-aluminosilicate complexes in the shoots of the plant. As a consequence of the increasing concentration the amount of Al transportation raised between the roots and shoots [113]. Moreover, Si is able to increase the Al tolerance of maize through phenolic compound metabolism, leading to more phenolics substances in the plant [114]. Catechin, quercetin, and other flavonoid-phenolics could potentially increase the heavy metal tolerance of plants.

#### 2.3.3. Silicon and Nutrient Imbalance

*(1) Silicon and Phosphorus*. Silicon is able to increase crop yield under P-deficiency stress. Supplying Si in nutrient solutions of rice resulted in an increase of rice shoot dry weight [115]. Although Si does not influence P accessibility inside the soil, the fixation capacity of P is not affected by the concentration of silicic acid in the soil [116]. It has been shown that P uptake by plants cultured in both solution and soil is not affected by Si treatment [115, 116]. Under P-deficiency, internal accessibility of P is controlled by other metals, such as Mn and Fe. Therefore, Si can increase P accessibility indirectly by decreasing the availability of Fe and Mn in plants [55].

Silicon can affect both deficiency and over dose stresses of P. Silicon is also able to suppress the damaging effects of excess P by decreasing extreme P absorption because Si deposition in root endodermal cells [117] may act as an apoplastic hindrance to decrease P uptake by roots.

*(2) Silicon and Nitrogen*. The Si accumulation in the leaf blades and stems of rice decreases the mutual shading and sensitivity of plants to diseases caused by high nitrogen availability. The occurrence of blast disease considerably decreased in the field after Si treatment, particularly when over dosage of N happened in soil with dense planting [118].

#### 2.3.4. Silicon and Climate Condition

Silicon effectively decreases rice (*Oryza sativa*) damage under environmental stresses including inadequate sunshine, low temperature, and typhoons [20]. On the other hand, high Si accumulation in rice leads to increasing the culm wall thickness and vascular bundles size, consequently increasing the stem strength [119] and decreasing lodging. The deposition of Si on the hull stops the loss of water and allows the plant to withstand strong winds [120]. Moreover, the yield of Si treated rice productivity is unaffected by inadequate sunshine and low temperatures. There is less damage from electrolyte leakage as a result of high temperature on the leaves of Si treated plants than on the leaves of untreated plants [77].

### 2.4. Silicon and Biotic Stresses

#### 2.4.1. Silicon and Plant Disease

Reportedly, Si is able to decrease the susceptibility of rice against sheath blight diseases [11, 121, 122]. Plant opal or glass and hard coating of SiO_2_ polymerisation in the plant cuticle layer is the possible mechanism for reducing disease susceptibility by Si [12]. The physical hindrance created by SiO_2_ enhances the incubation period in the leaf sheath of rice and results in impeding* R. solani* penetration to decrease the number and extension of sheath wounds. In comparison to the physical hindrance to early penetration, the lesion extension is a more important factor in terms of resistance to sheath blight disease, particularly in susceptible cultivars [123]. Silicon leads to increase the sheath blight resistance through creating a physical hindrance by SiO_2_ and reduce the intensity of disease. It has been speculated that Si is able to decrease the effect of sheath blight by motivating the defence mechanisms of the crops against pathogenesis, increase the amounts of phenolic components, and increase the activities of peroxidase, chitinases, polyphenoloxidase, *β*-1, 3-glucanases, and phenylalanine ammonia-lyase enzymes [12].

Several studies have reported the suppressive role of Si on rice blast disease caused by* Magnaporthe grisea* [121, 124, 125]. Silicon can reduce the intensity of blast disease in the leaf and the panicle during different growth stages. Reduction of the leaf lesions of rice after 96 hrs of inoculation with* M. grisea* between Si-treated and untreated plants has been examined by Rodrigues et al. [124]. The experiment results indicated numerous coalescing and large lesions, regularly surrounded by a chlorotic halo, were observed on leaves of the untreated plant. However, separate and tiny lesions that seemed to be restricted at the expansion step were observed in Si-treated rice. Moreover, leaves of the control plants presented strong chlorosis compared to the Si-treated plants.

The intensity of neck and leaf blasts in both sensitive and partially resistant rice cultivars can be decreased* via* Si treatment depending on the rate of Si application and the disease severity [126]. Superior inherent disease severity at specific sites needs a lot of Si fertiliser to decrease the neck and leaf blast disease as effectively as in resistant rice cultivars. The Si has been applied to prevent the occurrence of powdery mildew disease, one of the plant diseases created by* Sphaerotheca fuliginea*. Silicon has been reported to be an effective suppressor of powdery mildew (*Blumeria graminis*) [8, 127–129]. Increasing the Si content in cucumber shoots leads to a decrease in powdery mildew incidence [130]. Additionally, it has been reported that the occurrence of powdery mildew disease decreased after increasing the concentration of Si in the culture solution [131]. The macroelement can decline infection efficiency, colony size, and conidia germination in cucumbers [132]. Effective use of Si in a foliar approach has been reported to help the growth of leaves in grapes, cucumbers (*Cucumis sativus*), and muskmelons (*Cucumis melo*) [133, 134]. The growth trend most likely depends on the Si deposition on the surface of the leaves. Silicon also increases the tolerance of cucumber roots against* P. aphanidermatum *and* Pythium ultimum* fungal diseases [135]. Silicon also has prohibitive effect on rice green leafhoppers (*Eurymela distinct*), leaf spiders, brown plant hoppers [136], white­backed planthoppers, and mites (*Lorryia formosa*) [137]. Along with all the above, the resistance of rice to the brown plant hopper (*Nilaparvata lugens*) is related to the Si content of plant [136]. Furthermore, it has been document that Si is able to increase the tolerance of sorghum (*Sorghum bicolor*) to anthracnose [138].

## 3. Transportation and Deposition of Silicon

The Si absorption intensity is variable depending on the plant species. Mitani and Ma carried out a study on transportation of Si by three different plant species including rice (*Oryza sativa*), tomato (*Solanumly copersicum*), and cucumbers (*Cucumis sativus*) with different Si absorption capacities (high, medium, and low) and indicated that Si transportation between the exterior solution and the cell cortical is controlled by a particular transporter (*K*_*m*_ value of 15% mM) in all three species [39]. However, these three species have different *v*_max⁡_ from high to low, concluding that the transporter density differs between species. It has been speculated that Si transportation requires energy, and low temperature and metabolic repressors inhibit Si transportation [39]. Silicon transportation between cortical cells and the xylem is the most important factor which is responsible for high level of Si deposition in rice shoots. The lower Si deposition in some species, such as cucumbers (*Cucumis sativus*) and tomatoes (*Solanumly copersicum*), is related to lower Si transportation density between the exterior solution and the cortical cells or an imperfect Si transporter between the cortical cells and the xylem. Silicon absorption through xylem loading in rice is related to only one type of transporter. In comparison, transportation in cucumbers and tomatoes happens by different transporters. Following the Si absorption by the roots and translocation in the xylem, silica gel (SiO_2_·*n*H_2_O) is formed by the polymerisation of the high concentration silicic acid (>2 mM). The xylem sap in rice and wheat involves the extra Si concentration presented as monomeric silicic acid [43]. Water transpiration and Si polymerisation are the two main factors for producing a high concentration of silicic acid in plant shoots. By increasing the concentration of silicic acid, it is initially converted to colloidal silicic acid and after that to silica gel [54].

It has been reported that Si absorption and transportation in rice and maize with similar Si-accumulator mechanism and in other species like wax gourd and sunflower as intermediate species are dependent on both plant species and outer Si concentration. All in all, the impact of plant species on Si uptake and Si transport is related to the ability of silicon absorption in passive or active form [139].

It has been demonstrated that silicification occurs in endodermis part of roots of gramineae during maturation. However, the cell walls of other tissues including vascular, epidermal, and cortical may be silicified in older roots. Also silicification occurs in various parts of grasses such as roots and shoot including leaves and culms, largely in the inflorescence [140].

Results of an investigation on rice [141] showed that a layer of deposited Si (2.5 *μ*m) is formed instantly under the cuticle with a double layer of Si-cuticle in the leaf blades. The results also demonstrated that the silicification of cells including dumb-bell-shaped cells in vascular bundles, silica cells, and silica bodies on bulliform cells is not limited to the rice leaf blades because silicified cells can also be found within the epidermis layer and vascular tissue of leaf sheath, stem, and hull [141]. Results of other studies on* Equisetum* sp. revealed that the silicified structures were found on cell wall epidermal surface as discrete rosettes and knobs sheltered in spicules [142, 143]. The location of this silica surface has effects on its thickness such as 3–7 mm in stem and 0.2–1.0 in leaf of plants.

Nuclear magnetic resonance (NMR) technique and colorimetric method have been used to investigate Si uptake in wheat [144]. The NMR technique was able to detect precisely any molecular species comprising Si. However, this technique only detected two silicic acid species (monomeric and dimeric) in earliest exudates and was not able to measure soluble Si in the later exudates using ^29^Si-NMR spectroscopy.

The silicified cells produced in leaf blades, vascular and epidermis stem tissues, and hull and leaf sheath play a protecting role against a variety of stresses in plants and provide functional archaeological and paleoecological information [52]. High Si deposition in rice decreases the ability of the roots to absorb Si [5]. Silicon absorption by the roots of rice is much higher than the roots of maize (*Zea mays* subsp.* mays*), wheat (*Triticum* spp.), rye (*Secale cereale*), barley (*Hordeum vulgare* L.), and sorghum [53].

The initial study of a Si transporter gene was in marine diatoms in 1997 [145]. The transportation of Si in naked* Cylindrotheca fusiformis*, a marine diatom, is encoded by a gene family [146]. Results of a study on transferring a diatom Si gene to a tobacco without an observed increase in Si absorption showed that the Si uptake mechanism is different between higher plants and diatoms [147]. It was speculated that the high Si content observed in rice shoots is not related to silicic acid diffusion through the lipid of the cell membrane [148]. However, there must be an active Si transporter in the roots of rice leading to high Si accumulation in the shoots [148, 149]. The first active Si absorption gene (*Lsi1*) between higher plants has been identified in rice [53, 147]. Three transporter genes* Lsi1*,* Lsi2*, and* Lsi6 *that are used to absorb and transport Si were identified in rice [49, 150]. The* Lsi1 *was isolated from rice and was cloned, characterised, and functionally analysed in 2006 [151]. A* Lsi1* mutant was used to find and map the genes responsible for Si xylem loading in rice [147].

Both* Lsi1 *and* Lsi2 *are located on the cell plasma membrane of endodermis and exodermis root cells on the distal side and proximal side, respectively [150, 151]. The complementary DNA of* Lsi1 *is 1409 bp, and the deduced protein is 298 amino long. Expression of* Lsi1 *and* Lsi2 *has been observed constitutively in the roots of rice [151].* Lsi6 *is similar to* Lsi1 *and* Lsi2 *and consists of five exons and four introns with an 894 bp open reading frame (ORF). Like* Lsi1*,* Lsi6* encodes a protein of 298 amino acids.* Lsi6* is expressed in the leaf blades, leaf sheaths, and roots, while* Lsi1* and* Lsi2 *are only expressed in the roots. The predicted protein of both* Lsi1* and* Lsi6* consists of two well conserved NPA domains (Asn-Pro-Ala) and four transmembrane domains [34, 49]. In contrast to* Lsi2*, which acts as a silicic acid efflux transporter [150], both* Lsi1* and* Lsi6* genes are silicic acid influx transporters [49, 151, 152]. It has been reported that* Lsi1* and* Lsi2 *significantly increase the absorption of Si by roots [150], while* Lsi6 *is involved in transferring of Si in shoots [49].* ZmLsi1* and* ZmLsi6 *have been identified in* Zea mays *and are involved in Si absorption and transportation in different parts of the roots [153].* ZmLsi1* is expressed highly in the lateral roots and slightly in the crown roots. In contrast,* ZmLsi6* is more highly expressed on the sheathes and blades of leaf and crown root [153].

**Figure 5:**
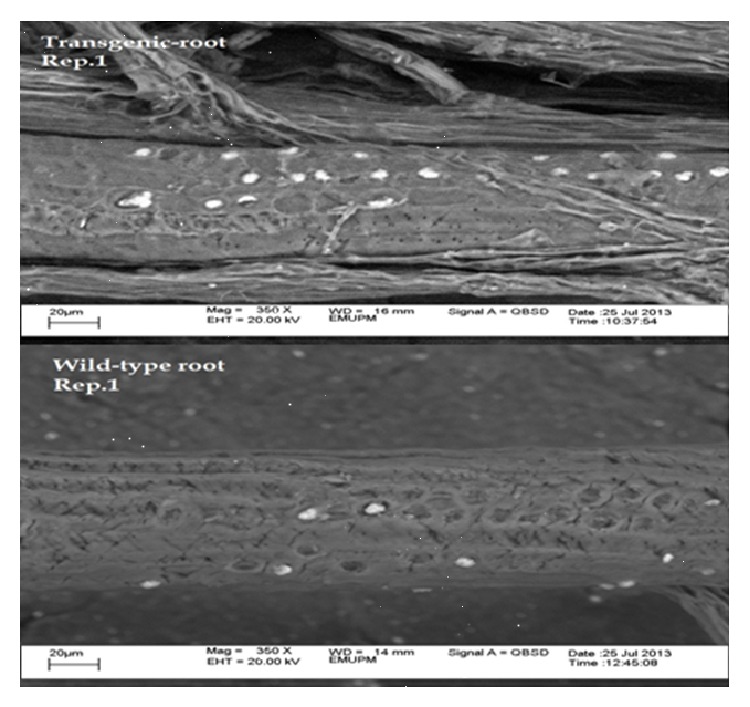
Electron microscopy image showing the Si accumulation (white spot) in transgenic and wild-type plant roots of* Arabidopsis thaliana*.

It has been speculated that* serine-rich proteins, proline-rich proteins,* and other polysaccharides are involved in Si accumulation [154]. Recently, the* serine-rich protein* gene was isolated from the roots of mangroves (*Rhizophora apiculata*) that is possibly responsible for Si absorption and accumulation in plants [24]. Computational analysis of the cDNA cloned and isolated from mangrove roots treated with different concentrations of Si in different periods of time through the suppression subtractive hybridisation (SSH) indicated that the* serine-rich protein* gene has a 696 bp coding region for a protein of 223 amino acids and is most likely involved in Si absorption and transportation in the roots of plants. The result of another survey [23] indicated that* serine-rich protein* gene increases amount of Si absorption and accumulation notably in the leaves and roots of transgenic* Arabidopsis thaliana* compared with wild-type plants (Figure 5). It can be suggested for future studies to provide some linkage between the polymerisation of amino acids-silicate and* serine-rich proteins*.

## 4. Biosilica Formation Mechanisms

Plant species, diatoms, and sponges are able to accumulate, store, and process Si to create an elaborated pattern of biosilicas. The silica production by organisms is formed at atmospheric pressure and temperatures ranging from 4 to 40°C in an aqueous phase of saturated silica solution [155]. The mechanism of biosilica formation by organisms has been the subject of vast research and discussions till today. It was reported that an organic environment, containing a wide range of carbohydrates, proteins, lipids, phenolic compounds, and metal ions, may play a primary role in biosilica formation [155, 156].

The silica condensation in the nature is affected by many factors including silica concentration, pH, temperature, and presence of other polymers, small molecules, and different ions. In all different silica species polymerization the angle of Si–O–Si bond and distances of Si–O bond play the fundamental role. The material involved in silica polymerization containing OH groups and environmental reactions may differ in different species because of the solubility, composition, hardness, viscosity, and density [157].

**Figure 6:**
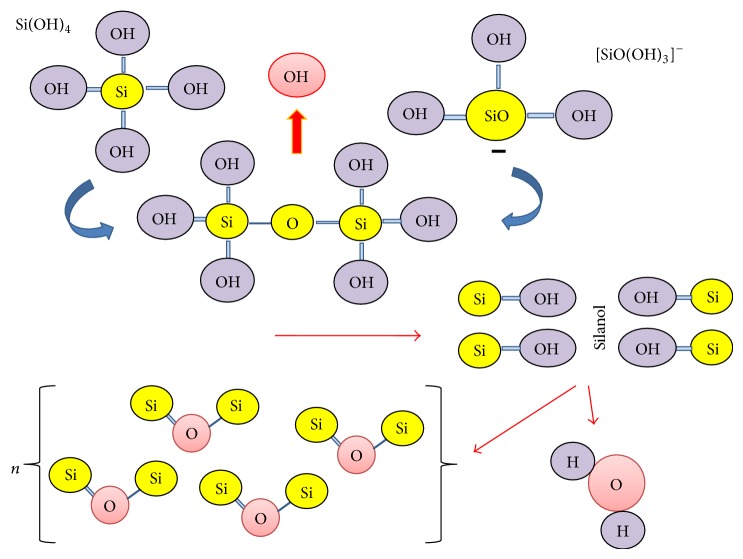
Schematic of Si species oxolation process.

Several* in vitro *and* in vivo *studies have been conducted to show the importance of biomolecules in biosilicification. Functional groups of all amino acids residues in protein's structure are accessible to silica and have a key role in determining the physical structure and nature of the substances which form during the maturing stages. It has been suggested that the role of amino acid in biosilicification may be the same as their* in vivo* role. In this case, effects of amino acids in biosilicification and their* in vivo* arrangements role can be a key factor to control biosilicification [158]. Peptides and amino acids are effective in creating polysilicic species* via* interactions with different species of silicate in the solution. Both forms of the silicate species, neutral Si(OH)_4_ and negatively charged [SiO(OH)_3_]^−^, are implicated in the oxolation procedure (Figure 6).

The oxolation process results in Si polymerisation by concentrating silanol units and releasing H_2_O molecules. The SN2 nucleophilic substitution or oxolation process involves transferring internal proton through the transition state leading to produce H_2_O molecules in leaving form. The SN2 nucleophilic substitution can be base or acid catalyzed and pH is the main determinative factor for the rate of oxolation reaction. Oxolation process is highly dependent on the pH. In case of silica it starts at pH 3, a minimum zero charge, and increased by increasing pH. The Si polymerisation rate increases at pH as high as 8-9. Hydrogen bonds with silicate species and the electrostatic at traction between the groups of (Si–O^−^ and –NH_3_^+^) are predicted to occur during oxolation (Figures 4 and 6). The p*K*_*a*_ of the amino acid side chains determines the ionisation in aqueous solution and creates the global charge.

**Figure 7:**
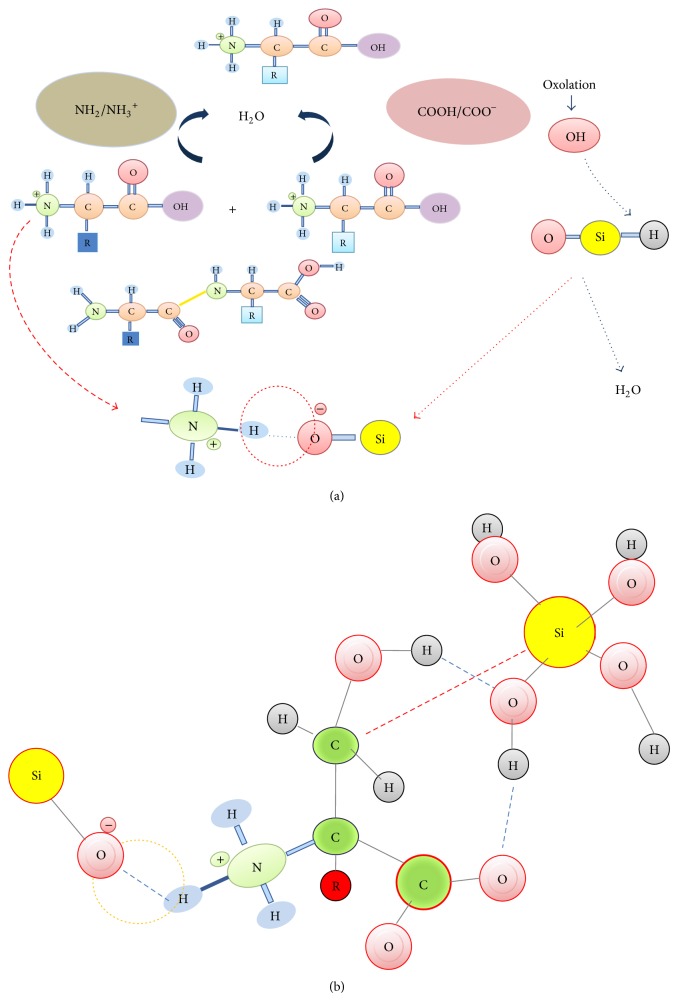
The putative relationship of the polymerisation of amino acids and silicate oxolation (a) and silicon (b). Blue dot row: hydrogen bond; red dot row: covalent bond.

The p*K*_*a*_'s of the amino and carboxylic groups of amino acids are approximately 9 to 10 and 2, respectively. Hence, at a neutral pH, the (COOH) group of amino acids are (COO^−^) or negatively charged, whereas the amino group or (NH_2_/NH_3_^+^) of the amino acids must be protonated. In a study conducted to show the effects of four amino acids (serine, proline, lysine, and aspartic acid) on polysilicate formation [159], the nucleophilic catalytic power of protonated amine groups was not observed. Furthermore, the number of NH_3_^+^ groups decreased by increasing pH, whereas it increased at the negative charge of the polysilicates. It was concluded that there must be some stability balance between these repercussions (Figure 7). The surface area of the silicate components may be affected by different amino acids depending on the hydrophobicity, the isoelectric point, and the silicate average pore size [158].

The results of another experiment showed that the carboxylic acid and alcohol groups of P-serine and P-asparagine amino acids did not have any significant role in the formation of the polysilicates, most likely due to the negative charge of these groups, on the other hand, the –OH side groups of serine appeared to be involved in hydrogen bond formation with Si(OH)_4_ [159]. However, it has been reported that the amine-terminated surfaces are not highlighted in silica nucleation; other substrates, such as carboxyl and hybrid NH_3_(^+^)/COO(^−^), are mainly active for silica accumulation. On the other hand, the free energy hindrances to forming the silica cluster are similar on both carboxyl- or amine-terminated surface forms [160]. Among all of the amino acids, p-Asp and p-Ser are impressive catalysts at pH 4.9, where the main silicate specie is neutral Si(OH)_4_. However, these amino acids are less energetic at pH 9.2, where the predominant silicate specie is negatively charged [SiO(OH)_3_]^−^ [159]. It has been shown that organic molecules containing hydroxyl-group are not involved chemically in the silica formation at all. Instead, they may only assist in rendering solubility and stability of the occluded organic molecules found in silica. In another view, it can be assumed that if hydroxyl-groups of organic molecules affect the silica formation, the polymerization of silicic acid would be encountered with water deficiency to increase the functional effects of protein's hydroxyl groups in the silica formation [161]. It can be predicted that the serine amino acid is appropriate for the oxolation process resulting in biosilica formation because this amino acid is neutral in terms of water solubility at different pH ranges and has a simple structure compared to other amino acids. As it is mentioned in the literature, the only form of Si that is absorbable by plants is monosilicic acid. Due to the dilution of the silicate solution, silanol molecules are not easily able to bond with amine groups. Therefore, the side groups of the amino acids and the carbonyl groups involved in the polypeptide chain are more likely to bring the molecules together, and biosilica formation occurs.

It can be understood that both amino acid and peptide can interact with silicate solutions* via* electrostatic interactions and hydrogen bonds. However, peptides seem to have a more significant effect than amino acids since hydrogen bonds or electrostatic interactions with a single molecule (amino acid) are not strong enough to a favorable condensation reaction. Since the condensation between molecular precursors occurs while species became close together it may be said that the distribution of hydrogen-bond (forming carbonyl groups) and charged side groups along the peptide chain brings species close to each other in the reactive. Moreover, the spontaneous collisions coming from Brownian motion are rather few in the silicate solutions [159].

## 5. Conclusions

The primary purpose of this review is to provide comprehensive insight about the role of Si in plants and the effects of biomolecules that are involved in the biosilica formation mechanism. Silicon plays an important role in helping plants overcome different types of abiotic and biotic stresses. The macroelement also improves the soil conditions under toxic levels of heavy metals and several chemical elements. In addition to the role of Si as a physical hindrance, the application of Si could affect the physiological and metabolic activities of plants. It is reasonable to recommend Si as a useful element involved in cellular processes. Understanding the roles of Si on higher plants may improve their growth and productivity yield and decrease their susceptibility to a wide range of diseases. Because of 3 hydroxypropanoic groups, serine is classified as a hydrophilic, polar amino acid. Serine plays an important role in the anabolism of pyrimidines and purines. The structure of serine helps this amino acid to participate in other metabolites by easily releasing one atom of carbon in biosynthesis. Hence, serine is an important component in the biosilica formation mechanism and improvement of plant metabolism. Most of plants, especially dicots, are not able to absorb large quantities of Si from the soil. Hence, genetically and biochemically manipulating the plant roots to increase their capacity of Si absorption and subsequently transferring of Si to the shoot parts could help plants to overcome a wide range of stresses and improve their metabolism.
